# Protective Effect and Possible Mechanisms of Artemisinin and Its Derivatives for Diabetic Nephropathy: A Systematic Review and Meta-Analysis in Animal Models

**DOI:** 10.1155/2022/5401760

**Published:** 2022-04-25

**Authors:** Haoyue Feng, Tingchao Wu, Qi Zhou, Hui Li, Tianyi Liu, Xitao Ma, Rensong Yue

**Affiliations:** ^1^Hospital of Chengdu University of Traditional Chinese Medicine, Chengdu, China; ^2^Chengdu Second People's Hospital, Chengdu, China; ^3^Chengdu University of Traditional Chinese Medicine, Chengdu, China; ^4^School of Acupuncture and Moxibustion, Hospital of Chengdu University of Traditional Chinese Medicine, Chengdu, China; ^5^Chongqing Fuling People's Hospital, Chongqing, China

## Abstract

**Background:**

Artemisinin and its derivatives have potential antidiabetic effects. There is no evaluation of reported studies in the literature on the treatment of diabetic nephropathy (DN), one of the commonest diabetic microangiopathies, with artemisinins. Here, we aimed to evaluate preclinical evidence for the efficacy and possible mechanisms of artemisinins in reducing diabetic renal injury.

**Methods:**

We conducted an electronic literature search in fourteen databases from their inception to November 2021. All animal studies assessing the efficacy and safety of artemisinins in DN were included, regardless of publication or language. Overall, 178 articles were screened according to predefined inclusion and exclusion criteria. Finally, 18 eligible articles were included in this systematic review. The SYstematic Review Center for Laboratory animal Experimentation (SYRCLE) risk-of-bias tool was used to assess the risk of bias in the included studies. The primary outcomes were kidney function, proteinuria, and renal pathology. Secondary endpoints included changes in fasting plasma glucose (FPG) levels, body weight, and relevant mechanisms.

**Results:**

Of the 18 included articles involving 418 animal models of DN, 1, 2, 6, and 9 used dihydroartemisinin, artemether, artesunate, and artemisinin, respectively. Overall, artemisinins reduced indicators of renal function, including blood urea nitrogen (*P* < 0.00001), serum creatinine (*P* < 0.00001), and kidney index (*P* = 0.0001) compared with control group treatment. Measurements of proteinuria (*P* < 0.00001), microalbuminuria (*P* < 0.05), and protein excretion (*P* = 0.0002) suggested that treatment with artemisinins reduced protein loss in animals with DN. Artemisinins may lower blood glucose levels (*P* = 0.01), but there is a risk of weight gain (*P* < 0.00001). Possible mechanisms of action of artemisinins include delaying renal fibrosis, reducing oxidative stress, and exerting antiapoptotic and anti-inflammatory effects.

**Conclusion:**

Available evidence suggests that artemisinins may be protective against renal injury secondary to diabetes in preclinical studies; however, high-quality and long-term trials are needed to reliably determine the balance of benefits and harms.

## 1. Introduction

Diabetes mellitus (DM) is a global public health challenge. The International Diabetes Federation Diabetes Atlas estimated that, in 2021, approximately 536.6 million adults aged 20-79 years were living with diabetes worldwide. By 2045, it is projected to reach 783.2 million people, with adults accounting for more than one-eighth of the total affected population [1]. The diabetes epidemic has also increased the incidence of diabetic nephropathy (DN), making it a leading cause of growing health problems and end-stage renal disease [2]. This ultimately results in 40% of patients requiring renal replacement therapy [3]. DN is a microvascular complication associated with glucose metabolism disorders, oxidative stress, and changes in renal hemodynamics [4, 5]. The early pathological features of DN include podocyte loss, glomerular hypertrophy, mesangial matrix expansion, and glomerular basement membrane thickening, while the later pathological features manifest as nodular glomerulosclerosis, mesangial lysis, and tubulointerstitial fibrosis [6, 7]. Therefore, early diagnosis and treatment can improve patients' quality of life and survival. Although the current strict glycemic control and management of renin-angiotensin system blockade have slowed the progression of DN, many patients with diabetes still progress to chronic kidney disease and eventually to end-stage renal disease [8], and the cardiovascular mortality of patients with diabetic kidney disease (DKD) continues to rise [9]. However, this first-line therapy for DKD has been considered unsatisfactory because of its potential side effects such as diabetic ketoacidosis [10] and reversible acute kidney injury [11]. Therefore, there is still an urgent need to find new treatments to prevent DN.

Artemisinin is a sesquiterpene endoperoxide derived from the Chinese herb sweet wormwood (*Artemisia annua* L.) and has been used as an essential antipyretic in Chinese medicine for thousands of years. The stability and easy crystallization of artemisinin render the extraction and purification process relatively simple; however, its therapeutic value is largely limited by its low solubility in both oil and water [12]. Thus, in the search for more effective and soluble drugs, researchers have prepared artemisinin derivatives, including artemether and artesunate, which are ultimately metabolizable to dihydroartemisinin (DHA). The efficacy and low toxicity of artemisinin and its derivatives for the treatment of malaria have been recognized. Following the awarding of the 2015 Nobel Prize for the discovery of artemisinin, this field has once again attracted people's interest and has promoted extensive research on the nonmalarial applications of artemisinin. These include biological effects against viruses, parasites, and tumors [13], as well as antifibrosis [14], antiarteriosclerosis [15], and potential hypoglycemic effects [16].

It is well known that artemisinins are cheap and have rapid activity, high potency, and minimal toxicity, but a short half-life, which makes artemisinins a candidate drug that maximizes the advantages of drug reuse [17]. Many studies have emphasized the potential of artemisinins as a novel antidiabetic agent. It is now understood that artemisinin and its derivatives slow down DM mainly by attenuating insulin resistance, improving immune microenvironment, and restoring islet cell function [16, 18, 19]. In addition, artemisinins have shown great promise for the treatment of diabetic complications, especially DN [20]. Current therapeutic tools for DN have limitations; artemisinin and its derivatives have the advantage of being multitargeted, and assessing their efficacy for DN treatment in animal models is of great significance for their future clinical translation. Recent findings suggest that artemisinins ameliorate DN by restoring mitochondrial function and inhibiting proliferation and combating fibrosis [21–23]. However, in the literature related to artemisinin treatment for DN, different investigators have focused on different indicators and reported differences in the efficacy of the same indicators. The scattered evidence, uncertainty of mechanisms, and adverse drug reactions add uncertainty and conflict to the hypothesis that artemisinins can ameliorate renal injury in diabetic animal model.

Systematic pooling and evaluation of available evidence is beneficial for finding a drug that combines efficacy and fewer side effects to prevent and treat DN. Artemisinin and its derivatives are not yet clinically available for the treatment of DN. A full review of the limitations and potential of all available evidence from animal studies prior to clinical trials would facilitate the translation of new therapeutic strategies from experimental results to the clinic. To our knowledge, this study is the first systematic evaluation of the renoprotective effects of artemisinin in DN. The key clinical questions we reviewed and addressed in our study included the following: (1) evaluation of the efficacy of artemisinins on specific indicators in animal models of DN, such as blood urea nitrogen (BUN), serum creatinine (SCr), and proteinuria; (2) assessment of the safety of artemisinins in DN; (3) review of the mechanisms of artemisinins in treating DN; (4) summarizing of the quality and limitations of existing animal studies; and (5) evaluation of the current evidence regarding whether artemisinins have clinical translational value for the treatment of DN.

## 2. Materials and Methods

### 2.1. Search Strategy

We conducted this report based on the Preferred Reporting Items for Systematic reviews and Meta-Analyses checklist. This study was registered with PROSPERO (registration number: CRD42021288364). The search included publications from 14 databases. The search time ranged from inception of the database to November 2021. Two authors independently searched the following electronic databases: PubMed, Embase, Web of Science, Scopus, Cumulative Index to Nursing and Allied Health Literature (CINAHL), OpenGrey, Google Scholar, Psyclnfo, British Library Ethos, ProQuest Dissertations & Theses, China National Knowledge Internet (CNKI), VIP Information Chinese Periodical Service Platform (VIP), China Biology Medicine Disc (CBM), and Wanfang Data Knowledge Service Platform (Wanfang), to determine animal reports on the use of artemisinin and its derivatives to treat kidney damage in diabetic nephropathy. There were no restrictions on the language or year of publication in this search. The PubMed database search mainly used the following term retrieval strategy: participants (“Diabetic Nephropathies,” “Nephropathies, Diabetic,” “Nephropathy, Diabetic,” “Diabetic Nephropathy,” “Diabetic Kidney Disease,” “Diabetic Kidney Diseases,” “Kidney Disease, Diabetic,” “Kidney Diseases, Diabetic,” “DN”), Intervention (“Artesunate,” “Artemisinins,” “Artemether,” “Artemisia annua,” “artelinic acid,” “artemisinine,” “artemotil,” “dihydroartemisinin”). The specific retrieval strategies used in the PubMed database are listed in Table S1. In addition, we appropriately modified some of the search terms to fit other databases.

### 2.2. Study Selection

The preestablished criteria for inclusion were as follows: (1) animal models of DN without restriction of species, sex, and modeling methods; (2) the treatment group received monotherapy with artemisinin and its derivatives at any dosage, timing, and frequency, and the control group received the same amount of nonfunctional substances or no treatment; and (3) the primary outcomes were BUN, SCr, kidney index (KI), proteinuria, microalbuminuria, urinary protein excretion, and pathological changes in renal tissue. The secondary outcomes were fasting plasma glucose (FPG) levels, body weight, and the mechanism of kidney damage caused by DM. The preestablished criteria for exclusion were as follows: (1) other animal models; (2) combined with other therapies; (3) without a separate control group; (4) studies in vitro, humans, or silico; (5) case reports, controlled studies with separate treatment groups, or crossover studies; (6) reviews; (7) duplicate publications; and (8) no predetermined primary outcome index.

### 2.3. Data Extraction

Two authors independently screened the retrieved studies based on inclusion and exclusion criteria. Initial literature screening was performed by reading the titles and abstracts, and the full text of the relevant studies was reviewed to assess their suitability for meta-analysis. Any differences were resolved by discussion with the corresponding author. Subsequently, an Excel form was created based on the following items: (1) first author and publication year; (2) details (sample size, species, age, sex, and weight) of animals; (3) method of establishing animal models and criteria for successful modeling; (4) types of artemisinins; (5) information regarding treatment and control groups (administration, dosage, and duration of treatment); and (6) outcomes and intergroup differences. For the result indicators displayed graphically, GetData Graph Digitizer software (version 2.26) was used to extract the data. To address the issue of the classification of the therapeutic drugs into subgroups in the original study, the Cochrane Handbook for Systematic Reviews of Interventions (CHSRI) [24] was chosen to combine the results of different subgroups into one treatment group for analysis.

### 2.4. Risk-of-Bias Assessment

Two researchers independently assessed the quality of the included studies using the SYstematic Review Center for Laboratory animal Experimentation (SYRCLE) risk-of-bias tool. The assessment content covered deviations in ten areas, and each item was scored as 1 point. Each item was as follows: (1) sequence generation, (2) baseline characteristics, (3) allocation concealment, (4) random housing, (5) blinding (for animal breeders and researchers), (6) random outcome assessment, (7) blinding (for outcome evaluator), (8) incomplete outcome data, (9) selective outcome reporting, and (10) other sources of bias. Any dispute arising from the evaluation was resolved by the corresponding author through negotiation.

### 2.5. Subgroup Analysis

We preset four subgroups to evaluate the influence of the variables or research characteristics on the estimated effect size: (1) modeling methods, (2) type of artemisinins, (3) route of administration, and (4) duration of treatment. In addition, subgroup analysis can be used to trace the sources of heterogeneity.

### 2.6. Data Synthesis and Analysis

RevMan software (version 5.3) was used for data analysis. As the main results were continuous variables, a standard mean difference (SMD) and 95% confidence interval (95% CI) were used to indicate the effect size. Heterogeneity was determined using Cochran's Q statistic and *I*^2^. *I*^2^ > 50% and *P*_*Q*−test_ < 0.1 suggested that there was significant heterogeneity, in which case the random effect model was used, whereas the fixed effect model was used. When significant deviations occurred in the individual results, a sensitivity analysis was performed. Potential publication bias was evaluated by Egger's linear regression test using Stata software (version 15.0).

## 3. Results

### 3.1. Study Selection

**Figure 1:**
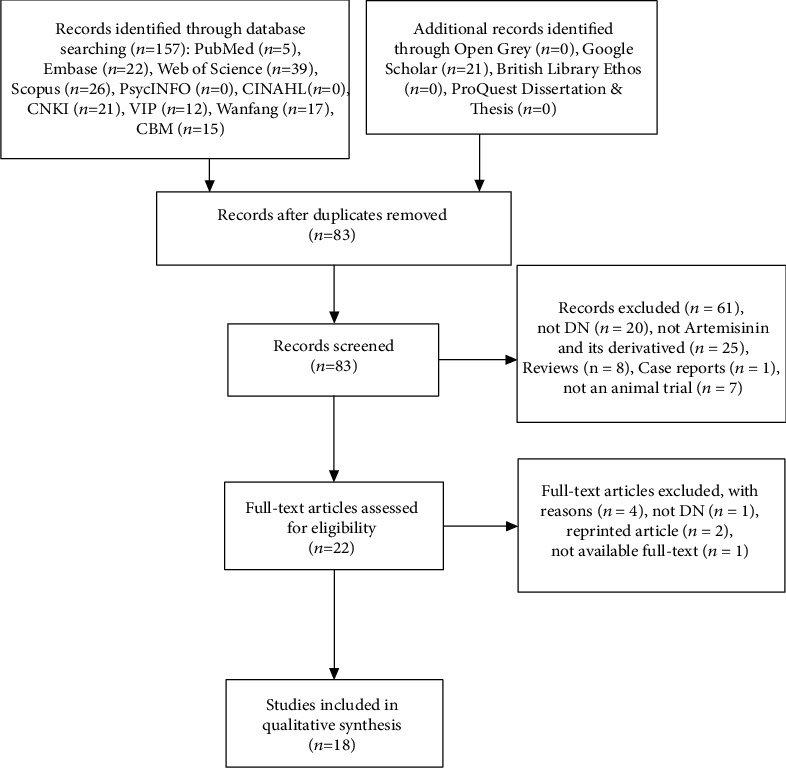
Flow diagram for selection of studies.

A total of 178 articles (5 from PubMed, 22 from Embase, 39 from Web of Science, 26 from Scopus, 21 from CNKI, 12 from VIP, 15 from CBM, 17 from Wanfang, and 21 from Google Scholar) were retrieved from online databases. Of these, 74 were duplicates. Fourteen studies were included in the systematic review and meta-analysis. The detailed selection process is illustrated in Figure 1.

### 3.2. Study Characteristics

Eighteen studies were published between 2014 and 2020 [21–23, 25–39]. In total, 418 DN model animals were enrolled in 18 studies (277 in the test group and 141 in the vehicle control group). All animal models included in the studies were rats or mice, including Sprague Dawley rats (SD rats) used in fifteen studies [22, 23, 25–29, 31, 33–39], Wistar rats used in one study [32], db/db mice used in one study [21], and C57BL/6J mice used in one study [30]. Male animals were included in 18 studies. Seven studies mentioned the age of the experimental animals, which ranged from five to eight weeks [21, 23, 26–28, 30, 36]. SD or Wistar rats weighed 160–300 g, C57BL/6J mice weighed 22–26 g, and no weight data was reported for db/db mice. Apart from the study using db/db mice (spontaneous mice), ten studies used models with streptozotocin (STZ) only [22, 30, 31, 33–39], six established the DN model by intraperitoneal injection of streptozotocin and high-fat diet [25–29, 32], and two performed excision of the right kidney in animal models [25, 29]. The STZ dose range was 30–65 mg/kg. Apart from the two studies that did not describe the modeling standard [21, 30], all other studies used blood glucose ≥16.7 mmol/L as the modeling standard. Artemether [21, 30], artesunate [22, 26–29, 32], DHA [23], and artemisinin [25, 31, 33–39] were administered in two, six, one, and nine studies, respectively. Nine studies used a dose gradient of artemisinin orally or intragastrically [22, 23, 25–29, 32, 36]. In terms of administration methods, three studies used intraperitoneal injections [33–35], two mixed the drugs into regular food [21, 30], and the remaining thirteen used oral gavage. Regarding outcome measures, 15 studies reported renal pathology [21, 22, 25–27, 29–31, 33–39], 11 reported BUN and SCr [22, 23, 26, 28, 31–36], 10 reported KI [22, 25, 26, 28, 29, 32–36], 6 reported urinary protein excretion [21, 30, 33–36], 5 reported proteinuria [25–27, 31, 32], 2 reported microalbuminuria [23, 29], 13 reported FPG [21, 25–36], and 10 reported body weight [21, 23, 25, 26, 28, 32–36]. Several studies have reported representative indicators of fibrosis such as transforming growth factor- (TGF-) *β*1 [22, 23, 25, 29, 36], SMAD [22, 23], E-cadherin [23, 25], and fibronectin [23, 25]. Some studies have reported relative changes in inflammation indicators, such as toll-like receptor 4 (TLR4) [26, 28, 32], interleukin-8 (IL-8) [28, 32], tumor necrosis factor- (TNF-) *α*, and monocyte chemoattractant protein-1 (MCP-1) [26, 27]. Some indicators of oxidative stress have also been reported, with superoxide dismutase (SOD) being the most frequent [21, 30, 36]. Detailed characteristics of the included studies are listed in Table 1.

### 3.3. Risk of Bias and Quality of Included Studies

Of the 18 included studies, three described the methods used to generate the allocation sequence [21, 26, 28], while the remaining studies lacked information about this process. Four studies [21, 26, 28, 30] reported similar baseline characteristics between the groups. No study clarified whether the allocation of different groups was sufficiently hidden. The breeding conditions and environment of all experimental animals included in the study were the same; therefore, we considered that the animal placement complied with the principle of randomization. No studies reported sufficient information regarding blinding methods for caregivers or investigators. In terms of randomization and blinding of outcome evaluation, all studies were unable to assess the exact risk. Two studies [21, 36] did not report complete outcome data. All prereported results were reported in all the included studies. Most of the studies had no other sources of bias. Due to the local intervention of the animal model in the way of modeling, two studies [25, 29] were judged to be high-risk. A complete quality assessment of the included studies is shown in Table S1.

### 3.4. Effects on Kidney Function

**Figure 2:**
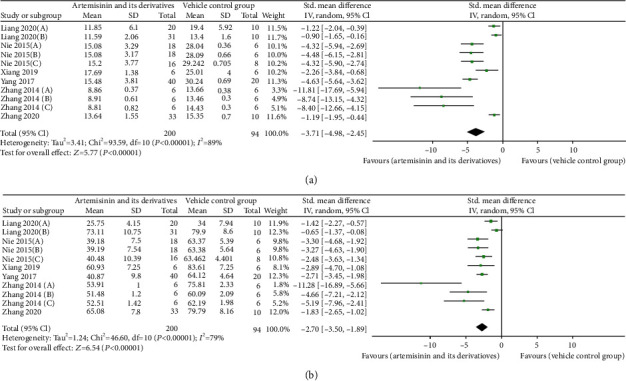
Pooled results of all trials examining the effect of artemisinins on (a) blood urea nitrogen (BUN) and (b) serum creatinine (SCr).

Eleven of the 18 studies (including 294 animals) provided data on the efficacy of artemisinin or its derivatives on BUN and SCr levels compared to treatment with a blank model group [22, 23, 26–28, 31–36]. Eleven studies reported that BUN was significantly lower in the artemisinins than in the control group (SMD: −3.71 [95% CI: −4.98, −2.45], *P* < 0.00001; heterogeneity: *I*^2^ = 89%, *P*_*Q*−test_ < 0.00001; Figure 2(a)). These same studies reported that artemisinins reduced SCr levels (SMD: −2.70 [95% CI: −3.50, −1.89], *P* < 0.00001; heterogeneity: *I*^2^ = 79%, *P*_*Q*−test_ < 0.00001; Figure 2(b)).

Through the stratified analysis of BUN and SCr, potential factors (including modeling methods, type of artemisinins, route of administration, and duration of treatment) that may have increased the heterogeneity of the results were explored. The analysis of BUN and SCr was repeated after stratifying the trials based on the modeling methods. Seven trials [22, 23, 31, 33–36] used the model induced by STZ injection alone, and pooled estimates showed a difference in BUN (SMD −2.76 vs. SMD −4.49, *P* < 0.05) but nonsignificant difference in SCr (SMD −2.69 vs. SMD −2.83, *P* > 0.05) compared to those of studies using the model induced by STZ injection combined with high-fat diet (HFD) [26–28, 32], but all studies had decreased heterogeneity compared to the previous studies.

The subgroup analysis results of SCr and BUN were similar in terms of the type of artemisinins, route of administration, and duration of treatment. Among the three drugs, artemisinin [31, 33–36], artesunate [22, 26–28, 32], and DHA [23], the effect size of artemisinin was better than that of artesunate and DHA (SMD −5.43 vs. SMD –3.74 vs. SMD −0.90, *P* < 0.05; SMD −4.16 vs. SMD –2.53 vs. SMD -0.65, *P* < 0.05), and the heterogeneity was slightly lower than before. No difference was seen between the short (≤8 weeks) and the long (>8 weeks) periods of artemisinins treatment [22, 23, 26–28, 31–36] (SMD −3.64 vs. SMD −4.32, *P* > 0.05; SMD −2.76 vs. SMD −2.48, *P* > 0.05). In terms of the route of administration, subgroup analysis showed that the effect of intraperitoneal injection [33–35] was better than that of intragastric administration [22, 23, 26–28, 31, 32, 36] (SMD −9.26 vs. SMD −2.82, *P* < 0.05; SMD −6.10 vs. SMD −2.20, *P* < 0.05). The results of the subgroup analyses for BUN and SCr levels are presented in Table 2 and Item S2.

### 3.5. Effects on Proteinuria

**Figure 3:**
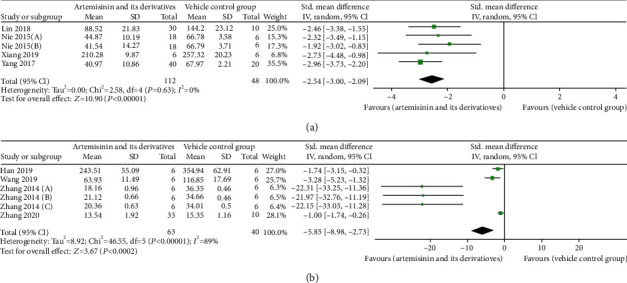
Pooled results of all trials examining the effect of artemisinins on (a) proteinuria and (b) urinary protein excretion.

The effect of artemisinins on the ability to reduce proteinuria was assessed in five trials [25–27, 31, 32]. In these studies, a meta-analysis suggested that, although the magnitude of proteinuria reduction varied across artemisinin and its derivatives, effect point estimates showed potential benefits for DN (*n* = 160; SMD: −2.54 [95% CI: −3.00, −2.09], *P* < 0.00001; heterogeneity: *I*^2^ = 0%, *P*_*Q*−test_ = 0.63; Figure 3(a)). Only microalbuminuria was described because the number of studies was too small (<3 studies). In the two studies, artemisinins were found to reduce microalbuminuria compared to the treatment in the control group (*P* < 0.05). In the other six studies [21, 30, 33–36], 24-hour urine collection was performed to assess urinary protein excretion. When all data were combined, there was evidence of heterogeneity between studies (*I*^2^ = 89%, *P*_*Q*−test_ < 0.00001), and the SMD for the treatment effect of artemisinins on proteinuria was statistically significant (*n* = 103; SMD: −5.85 [95% CI: −8.98, −2.73], *P* = 0.0002; Figure 3(b)).

Proteinuria was not combined and analyzed in subgroups because of the different criteria and small number of studies. However, by reanalysis of the urinary protein excretion, we learned that artemisinin had better renal function protection compared with artemether (SMD −2.35 vs. SMD −16.29, *P* > 0.05; Item S2).

### 3.6. Effects on Renal Pathology

**Figure 4:**
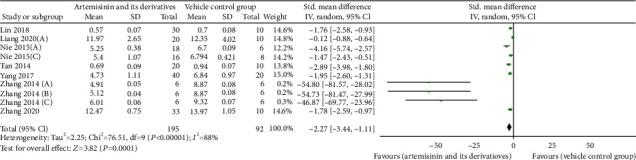
Pooled results of all trials examining the effect of artemisinins on kidney index (KI).

The KI is the ratio of kidney weight to body weight. Usually, the ratio of kidney to body weight is relatively constant. When a kidney is damaged, its weight changes, and so does the renal coefficient. An increase in the renal coefficient indicates congestion, edema, or hypertrophy of the organ, while a decrease indicates degenerative changes, such as renal atrophy [40]. All 10 studies [22, 25, 26, 28, 29, 32–36] reported an increase in KI in the DN model, while the use of artemisinins resulted in a significant decrease in the KI value (*n* = 287; SMD: −2.27 [95% CI: −3.44, −1.11], *P* = 0.0001; heterogeneity: *I*^2^ = 88%, *P*_*Q*−test_ < 0.00001; Figure 4).

Thirteen studies [21, 22, 25–27, 30, 33–39] reported that artemisinins significantly alleviated membrane cell proliferation and broadening of the membrane matrix compared to treatment in a control group. Treatment with artemisinins inhibited thickening of the substrate membrane in 11 studies [21, 26, 27, 30, 33–39]. There was a reduction in enlarged glomerular volume in eight studies [21, 29–31, 35, 37–39], significantly ameliorated foot process effacement in three studies [21, 30, 33], and alleviated glomerular fibrosis in one study [22]. At the same time, periodic acid-Schiff (PAS) staining of the glomerulus and renal tubule was ameliorated (*P* < 0.05) in one study [21], and PAS staining of the glomerular capillary area and proximal tubular area was reduced (*P* < 0.05) in another [30].

### 3.7. Effects on FPG and Body Weight

**Figure 5:**
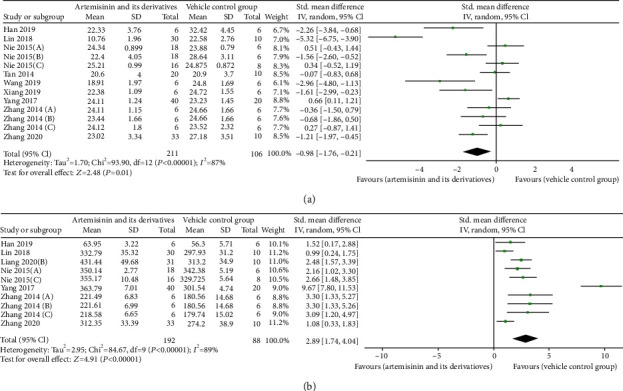
Pooled results of all trials examining the effect of artemisinins on (a) fasting plasma glucose (FPG) and (b) body weight.

Data on the effects of artemisinins compared to those of the control group treatment on FPG levels were available from 13 trials [21, 25–36], including 317 animals. Overall, treatment with artemisinins reduced FPG levels compared to the treatment with the control. There was significant heterogeneity in the extent of the effect in these experiments (*I*^2^ = 87%, *P*_*Q*−test_ < 0.00001). Again, the advantage of this benefit was not significant (SMD: −0.98 [95% CI: −1.76, −0.21], *P* = 0.01; Figure 5(a)).

The effects of artemisinins on body weight levels were reported in 10 trials [21, 23, 25, 26, 28, 32–36]. Analysis of these studies showed that body weight levels were higher in the artemisinin than in the control groups (*n* = 280; SMD: 2.89 [95% CI: 1.74, 4.04], *P* < 0.00001; heterogeneity: *I*^2^ = 89%, *P*_*Q*−test_ < 0.00001; Figure 5(b)).

### 3.8. Kidney Protective Mechanisms

#### 3.8.1. Antifibrosis

**Figure 6:**
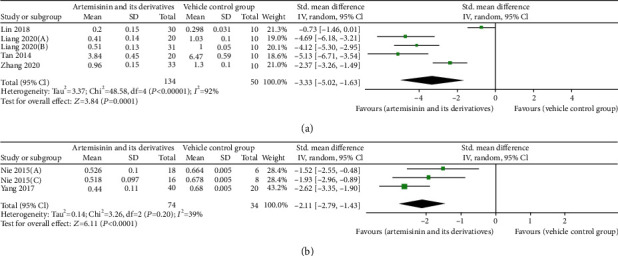
Pooled results of all trials examining the effect of artemisinins on (a) tumor growth factor-*β*1 (TGF-*β*1) and (b) toll-like receptor 4 (TLR4).

Five studies reported the effect of artemisinins on the TGF-*β*1 protein [22, 23, 25, 29, 36]. Results from the TGF-*β*1 meta-analysis indicated that the intervention group had reduced TGF-*β*1 protein levels compared with the control group in DN animals (*n* = 184; SMD: −3.33 [95% CI: −5.02, −1.63], *P* < 0.00001; heterogeneity: *I*^2^ = 92%, *P*_*Q*−test_ = 0.0001; Figure 6(a)). Two studies showed that artemisinins significantly lowered SMAD activity [22, 23], including SMAD2, SMAD3, and p-SMAD3. Two studies reported higher levels of E-cadherin and lower levels of fibronectin [23, 25], while one reported lower levels of ras-homolog gene family, member A (RhoA), Rho-associated coiled-coil containing protein kinase 1 (ROCK1), and *α*-smooth muscle actin (*α*-SMA) [25]. One study reported lower levels of connective tissue growth factor (CTGF) [29], and one reported higher levels of matrix metalloproteinase-2 (MMP-2) and lower levels of tissue inhibitor of metalloproteinases-2 (TIMP-2) [35].

#### 3.8.2. Anti-Inflammatory Cytokines

Three studies have reported the effect of artemisinins on the TLR4 protein [26, 28, 32]. Results from the TLR4 meta-analysis indicated that TLR4 protein levels were reduced in the intervention compared with the control group in DN animals (*n* = 108; SMD: −2.11 [95% CI: −2.79, −1.43], *P* = 0.20; heterogeneity: *I*^2^ = 39%, *P*_*Q*−test_ < 0.00001; Figure 6(b)). Two studies showed that artemisinins reduced IL-8 activity [28, 32]. One study reported significantly lower nuclear factor kappa B (NF-*κ*B) levels [29]. TNF-*α* and MCP-1 are expressed at lower levels in both kidney tissue and peripheral blood [26, 27].

#### 3.8.3. Antioxidation

Three studies have reported the effect of artemisinins on SOD protein levels [21, 30, 36]. One [21] reported no significant difference in the relative expression level of SOD2 protein between the artemisinins and control groups (*P* > 0.05). Another [36] reported the positive effects of artemisinins on malondialdehyde (MDA) and glutathione peroxidase (GSH-Px) levels, as well as a higher level of nuclear factor-erythroid factor 2-related factor 2 (Nrf2) and its downstream signaling molecules, including NAD(P)H quinone oxidoreductase-1 (NQO-1) and heme oxygenase-1 (HO-1).

#### 3.8.4. Other Renoprotective Mechanism

The included studies also reported the regulation of artemisinins by other proteins. These reports indicated that artemisinins had a positive effect on other mechanisms of kidney injury. These key proteins included mitochondrial pyruvate carrier (MPC) 1/2 [21, 30], AMP-activated protein kinase (AMPK) [23], phospho-protein kinase B (p-Akt) [30], phospho-mammalian target of rapamycin (p-mTOR) [30], and protein kinase C (PKC) [34]. All reported proteins are listed in Table 1.

### 3.9. Sensitivity Analysis and Publication Bias

We conducted a sensitivity analysis of the two primary outcome indicators, SCr and BUN. This analysis showed that deleting data from each trial had no significant effect on the overall heterogeneity of the results. After excluding each trial from the meta-analysis, there was no substantial difference between the sensitivity of the pre- and postsensitivity pooled effects. After ignoring studies by Zhang et al. 2014 (A) [33] and Liang et al. 2020 (B) [23], the lowest and highest pooled effects of BUN were −3.40 (95% CI: −4.62, −2.17) and −4.13 (95% CI: −5.53, −2.73), respectively; for SCr, they were -2.49 (95% CI: −3.22, −1.76) and −2.89 (95% CI: −3.63, −2.14), respectively.

**Figure 7:**
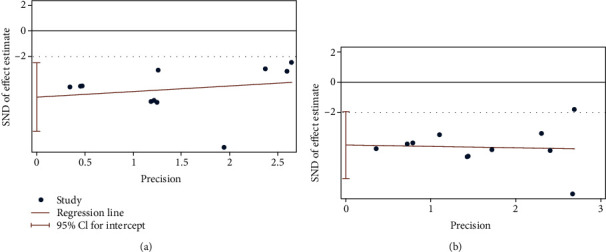
Egger's publication bias plot for (a) blood urea nitrogen (BUN) and (b) serum creatinine (SCr).

**Figure 8:**
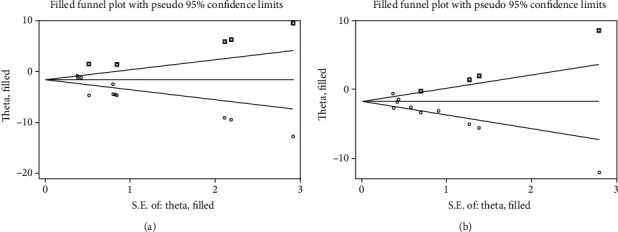
Trim-and-fill analysis for (a) blood urea nitrogen (BUN) and (b) serum creatinine (SCr).

BUN and SCr are core indicators of renal function. We used Egger's test to evaluate publication bias for both. The results showed a publishing bias in both observations (Figures 7(a) and 7(b)). The trim-and-fill method was used to assess the effect of publication bias on the results (Figures 8(a) and 8(b)). The results based on the random-effects model suggested that the two results were consistent with the pretrimming and filling results, indicating that publication bias did not affect the stability of these results (Table 3).

## 4. Discussion

### 4.1. Summary of Evidence

The results of this systematic review and meta-analysis of 18 preclinical studies indicate that artemisinin plays a beneficial role in treating DN in animals. We regarded artemisinin and its derivatives as the same intervention; by summarizing and analyzing the data from all the outcome indicators, we found that artemisinins could improve renal function indicators such as BUN, SCr, and proteinuria and reduce KI and pathological changes in renal tissue. The potential causes of these protective effects may be closely related to delayed fibrosis, anti-inflammatory, antioxidative stress, and increased renal autophagy. However, the results of major renal function indicators, such as BUN, SCr, and proteinuria, showed a high degree of heterogeneity in our meta-analysis. According to the results of the subgroup analysis of this study, the heterogeneity originated from the modeling methods, type of artemisinins, and route of administration. Therefore, more high-quality studies with larger sample sizes should be conducted to confirm our findings. In addition, given the publication bias of the two metrics, BUN and SCr, we used the trim-and-fill method to infer five potential studies missing BUN and SCr, respectively. The adjusted results were not significantly altered, suggesting that publication bias did not affect the stability of the results.

### 4.2. Limitations

This study has some limitations. First, despite subgroup analysis, the high heterogeneity of BUN, SCr, and proteinuria could not be ignored. Modeling methods, type of artemisinins, and route of administration differed among the indicators. This heterogeneity may have reduced the validity of the results. Second, because of the uncertainty in many risk and quality assessment items, significant sources of bias reduced the overall quality of evidence in this study and may have affected our conclusions. Third, although we searched exhaustively in the corresponding database, only 18 papers were included in this study and some of the indicators did not have adequate sample sizes. Fourth, details on key measures of randomization and blinding and other indicators of study quality, such as baseline levels of the included studies, were missing in many of the included studies. Therefore, some studies may have overestimated the effect of artemisinins, which may have influenced the results of our meta-analysis. Fifth, short-term trials and lack of reported side effects limited our assessment of the long-term tolerability of artemisinins. Sixth, angiotensin-converting enzyme inhibitors (ACEIs) or angiotensin receptor blockers may have a positive effect on DN, but only four of the papers included in this study compared artemisinins with such drugs. Finally, we found no studies using animals with hypertension or other diabetic comorbidities, which are factors that are also important in the development of DN and may have resulted in overestimation of the role of artemisinins in the treatment of DN.

### 4.3. Implications

Poor animal study design may be a key factor contributing to higher interstudy heterogeneity and a barrier to translating animal studies into clinical applications for potential human disease drugs. Therefore, we suggest that future preclinical studies follow the Animal Research: Reporting of In Vivo Experiments (ARRIVE) [41] or Harmonized Animal Research Reporting Principles (HARRP) [42] standards. Such specifications can effectively help researchers improve the quality of animal experiments and increase the reliability of results. To assess the therapeutic effect of artemisinins, we believe that animal models with comorbidities, as well as positive control groups, should be used in future studies. In addition, since almost all evidence was obtained in rats and mice, it is unclear whether the dose and treatment duration of artemisinins are effective in all species, including humans. Large animals, such as rhesus monkeys, have metabolic rates comparable to those of humans, and subsequent studies should be appropriately considered in such animals. The use of male animals in all preclinical studies is also a flaw in the experimental design. This sex preference in preclinical studies may also lead to unpredictable differences in efficacy in clinical applications and even the potential toxicity of artemisinins in female patients. Female animals are less sensitive to STZ than males, which may be related to the interference of estrogen with STZ action [43]; however, this issue can be addressed by increasing the STZ dose [44]. We suggest that future studies select experimental animals and modeling methods according to the specific purpose of the experimental design and the actual situation of clinical translation.

The subgroup analysis in this study suggested that differences in modeling methods were also a source of heterogeneity. There are various methods for establishing animal models of DN. For nonspontaneous models, STZ alone and STZ combined with HFD are the most commonly used modeling methods for DN. We observed a large variation in the use of STZ among the included studies. Studies have shown significant tubular necrosis and nephrotoxicity following STZ administration at a dose of 65 mg/kg [45]. Six of the included studies [33–35, 37–39] used 65 mg/kg of STZ, while another report suggested that diabetes was induced by 55 mg/kg of STZ without any renal toxicity [46]. We recommend that STZ be used with caution and that the dose chosen should be consistent with that which induces diabetes without significant renal damage.

In this meta-analysis, BUN and SCr levels were highly heterogeneous. The difference in the efficacy of different artemisinins may be one of the reasons for this. Due to differences in dose settings across studies and the effects of various experimental conditions, we combined multiple-dose groups into a single-dose group, but information on the dose-response relationship may be ignored. We have carefully read the existing literature to clarify the effect of dose on efficacy. Each of these studies reported that both artemisinin [36] and DHA [23] reduced BUN and SCr levels in a dose-dependent manner. Five studies [22, 26–28, 32] reported that different doses of artesunate reduced BUN and SCr levels, but two studies [26, 27] showed that there was no significant difference between the middle-dose (20 mg/kg) and high-dose (30 mg/kg) groups (*P* > 0.05). We found that continuously increasing doses of artesunate [36] (100 mg/kg) and artemisinin [31] (300 mg/kg) did not achieve the desired effect. Such a large oral dose range yielded similar results, suggesting that artemisinins may have low permeability and bioavailability. Previous studies have reported that artemisinin and its derivatives exhibit poor solubility, low bioavailability, and high first-pass effects [47]. With advancements in pharmaceutical technology, many new technologies have been applied to the formulation of artemisinin and its derivatives, providing possible avenues for the multifaceted therapeutic effects of artemisinins. Microemulsion [48] and liposome [49] technologies can enhance the stability of artemisinins in solution, increase solubility, and improve drug bioavailability. Transdermal modes of drug delivery, such as pressure-sensitive adhesive patches [50] and dissolving microneedles [51], can avoid hepatic and gastrointestinal first-pass effects and reduce the gastrointestinal adverse effects of artemisinins. The development and use of injections may also effectively solve the problem of intestinal absorption of artemisinins [52]. The subgroup analysis in this study showed that the route of intraperitoneal injection was indeed significantly better than intragastric absorption in studies using artemisinin. The results of another subgroup analysis showed that a longer treatment duration (>8 weeks) did not achieve more prominent efficacy. This may be related to the accelerated irreversible renal decline in diabetes.

Changes in proteinuria and urinary protein excretion have been widely used as indicators to evaluate the progression of renal disease, and the reduction of proteinuria has been suggested as a separate therapeutic goal in patients with diabetes. Although 11 studies [21, 25–27, 30–36] incorporating proteinuria and urinary protein excretion were pooled, insufficient detail was provided to extract outcome data based on baseline levels. Microalbuminuria is an early sign of DN, and two studies [23, 29] reported a positive effect of artemisinins on microalbuminuria. In this analysis, high doses of artemisinin [25] had additional antiproteinuric effects compared with the use of ACEIs, and artesunate [26–28] was also equal or superior to ACEIs in both antiproteinuric effects and improved renal function. A large population-based cohort study [53] showed that long-term ACEI use was associated with an increased risk of lung cancer. This may be related to the fact that ACEI use leads to the accumulation of bradykinin and substance *P* in lungs. If this result is confirmed, drugs such as artemisinins that show better efficacy in DN without oncogenic side effects deserve more attention.

Four artemisinins used for the treatment of DN were recorded in this study. At present, artemisinin and artesunate are considered potential candidates for the treatment of DN. Artemisinin was the most used of the artemisinins in the included studies (9 studies). Artemisinin has a fast onset of action and low toxicity and is rapidly absorbed into the gastrointestinal tract after oral administration, with a half-life of 2–5 hours, and approximately 80% of the drug is excreted in the urine and feces within 24 h of administration [54]. The increase in reactive oxygen species levels caused by hyperglycemia is at the core of the pathogenesis of DN [55]. Previous reports have shown that pomegranate [56], green tea [57], and resveratrol [58] can exert antioxidative stress effects in diabetes. The study found that artemisinin can protect against renal damage in DN by inhibiting the expression of TGF-*β*1 protein in kidney tissue, activating the Nrf2 signaling pathway, and enhancing the expression of antioxidant proteins [36]. The anti-inflammatory effect of artemisinin has been widely recognized, mainly through the regulation of NF-*κ*B activity in DN [39]. The antifibrotic effect of artemisinin was thought to be related to the downregulation of TGF-ß1 and *α*-SMA, upregulation of E-cadherin protein in renal tissue, and reversal of renal tubular epithelial cell transdifferentiation [25]. Artesunate is an artemisinin derivative that has been manipulated pharmacologically and is characterized by a shorter half-life (<1 h) [54]. Analogously, some studies have shown that artesunate also has anti-inflammatory [26–28, 32] and antifibrotic [22, 29] effects. Other artemisinin analogs, including DHA and artemether, were also reported in our study, but the number of studies in which they were used was relatively small, and their effects were lower than those of artemisinin and artesunate, while other analogs such as arteether and artificial ether were rarely or not reported at all. Therefore, exploration of the optimal range of efficacy and the most appropriate dosing regimen for artemisinin and artesunate needs to be clarified in future studies so as to further promote the use of artemisinins in DN. We also suggest that the efficacy evaluation of other analogs will continue to improve in future experiments.

Poor glycemic control is a major risk factor for the development and progression of DN. Studies have shown that intensive glycemic control can reduce the risk of microalbuminuria and macroalbuminuria [59]. If blood glucose levels improve, this may indirectly reflect the potential role of drugs in renal protection. Li et al. and Bai and Fu [16, 60] found that artemisinin could enhance gamma aminobutyric acid signaling to promote the conversion of islet cells to functional b cells, which may have potential for the treatment of diabetes. However, a recently published meta-analysis [61] showed that the effect of artemisinin and its derivatives on blood glucose were not significant. The results of the present study similarly showed that, although the FPG of the animals in the artemisinins group decreased compared to that in the control group, these animals remained in a hyperglycemic state. Therefore, the effect of artemisinins on DN may not be related to lowering blood glucose levels. The effect of artemisinins on body weight is also of interest. The results of this meta-analysis showed that the body weight of animals treated with artemisinins was significantly higher than that of the control group treatment. Body mass index is strongly associated with development of DN. However, the use of all four drugs (artemether, artesunate, artemisinin, and DHA) suggested a possible increase in body weight. Among these, artesunate had the greatest effect on body weight. This phenomenon has also been reported in rat and mouse models of other diseases. Significant weight gain was observed in the asthma animal model stimulated by DHA in the ovalbumin group [62]. In a 5% dextran sulfate sodium- (DSS-) induced ulcerative colitis mouse model, weight gain was observed after treatment with artemisinin and its analogs such as DHA [63], artesunate [64], artemisinin [65], and *β*-aminoarteether maleate (SM934) [66]. However, it was noteworthy that the use of artemisinin in normal mice did not show an increase in body weight [65], while colitis also resulted in weight loss, making it difficult to determine whether the disease improvement or the effect of the drug caused the weight gain. Species differences might also play a role in this process. Weight loss following artemisinin-piperaquine tablets was reported in a subacute toxicology study in rhesus monkeys [67]. No researchers have specifically reported the effects of artemisinins on body weight in adults. Although we have previously found that artemisinin has a hypoglycemic effect, weight gain has a detrimental effect on metabolic diseases, such as diabetes and metabolic syndrome, which is an important factor in assessing whether artemisinins are suitable for diabetes and its complications. In view of possible species differences, observation of body mass index should be given more attention in human trials.

### 4.4. Safety and Toxicity of Artemisinin Administration

In our study, there was no discourse on artemisinins toxicity. In previous studies, artemisinin and its derivatives have been suggested to have some toxic effects, including neurotoxicity, genotoxicity, hematologic and immunotoxicity, cardiotoxicity, nephrotoxicity, and allergic reactions, but these were closely related to dosage, duration, and diseases [68]. Animal and human studies have shown that long-term peak concentrations of artemisinins can lead to toxicity more readily than short-term treatments, which may partly explain why long-term treatment does not achieve greater efficacy [69]. Therefore, the specific nodes of drug onset and toxic effects are also a focus for future studies. Further exploration of the long-term efficacy and safety of artemisinins can also help to observe and reflect on the limitations of artemisinins in the treatment of DN and allow for better translation into drugs suitable for human use.

### 4.5. Possible Cellular and Molecular Mechanisms of Action of Artemisinins against DN

A systematic review of preclinical research can provide important insights for determining the direction for elucidating the mechanism in follow-up research. The possible renal protection mechanisms mediated by artemisinins are summarized as follows: (1) mediating renal fibrosis by inhibiting the expression of TGF-*β*1 and downstream signaling molecule (SMAD2 and SMAD3) levels to reduce the degree of a-SMA, CTGF, and fibronectin and increasing the degree of E-cadherin protein, thus inhibiting epithelial-mesenchymal transition of renal tubular epithelial cells by regulating the RohA/ROCK pathway; (2) antioxidant action by increasing activities of GSH and SOD to reduce the content of MDA, thereby activating the Nrf2 to initiate the transcription and expression of NQO-1 and HO-1; (3) improving matrix deposition and glomerulosclerosis by regulating the expression of MMP-9 and TIMP-1 to inhibit mesangial cell proliferation and extracellular matrix accumulation; (4) ameliorating kidney inflammation by inhibiting NF-*κ*B and TLR4 signaling pathways, which mainly suppress the expression of inflammation-promoting cytokines, including TNF-*α*, IL-8, and MCP-1; (5) reducing the renal enlargement by inhibiting Akt/mTOR and mitogen-activated protein kinase/extracellular signal-regulated kinase (MAPK/Erk) pathways, and downregulation of p-p27^Kipl^ protein; and (6) promoting renal autophagy and improving renal function by increasing the expression levels of 1A/1B-light chain 3 (LC3) and AMPK.

High glucose levels cause mitochondrial dysfunction, which is the initiating factor for DN. Two of the included studies reported the effects of artemether on mitochondrial function in animal models of DN. However, the results of the two studies [21, 30] were inconsistent; this may be related to the different animal models selected, and more studies are needed to confirm it. In addition, a study [31] reported the use of high-throughput analysis to help elucidate the main targets and important pathways of artemisinins as anti-DN agents. The antifibrotic effect of artemisinins on DN has been confirmed in several other studies [22, 23, 25, 29, 36]. Other enriched Kyoto Encyclopedia of Genes and Genomes (KEGG) pathways of differentially expressed genes provide directions for future molecular mechanisms and effects, including “glycine, serine and threonine metabolism,” “complement and coagulation cascades,” “p53 signaling pathway,” and “peroxisome proliferator-activated receptor signaling pathway.” This may be the next step in the direction for research into the mechanisms of action. This range of biological effects is considered strong evidence for the possible conversion of artemisinins to a nephroprotective agent.

### 4.6. Conclusion

In conclusion, our review suggests that artemisinin and its derivatives may improve renal function and proteinuria in animals with DN and may play a role in reducing the burden of renal dysfunction secondary to diabetes. Taken together, the protective mechanism of artemisinins against DN may be related to antioxidant, anti-inflammatory, and antifibrotic effects. Although artemisinins have been widely used in patients with malaria, no clinical studies have reported their use in DN. Large-scale, long-term, and high-quality trials are needed to confirm these findings before they can be applied to humans. In addition, weight changes should be documented in ongoing clinical studies on artemisinins in other diseases, as this is critical for accurately determining the merits of using artemisinins in metabolic diseases.

## Figures and Tables

**Table 1:** Basic characteristics of included studies.

Study	Species (sex; n = treatment/control group), age/weight	Model		Experimental group	Control group	Administration/duration	Outcomes	Intergroup differences
		Establish	Modeling standard					
Han et al., 2019	db/db mice (male, 6/6), 8 weeks/NM	Spontaneous diabetes	NM	Regular diet supplemented with artemether, 670 mg/kg/d	Regular diet	By free access to the diets, 12 weeks	(1) Protein excretion (2) Body weight (3) FPG (4) Catalase↓ (5) SOD2↓ (6) PGC-1*α*↑ (7) PDK1↑ (8) PDH↑ (9) MPC2↑ (10) MPC1↑ (11) PDP1↑ (12) Renal pathology	(1) *P* < 0.05 (2) *P* < 0.05 (3) *P* < 0.01 (4) *P* > 0.05 (5) *P* > 0.05 (6) *P* < 0.05 (7) *P* > 0.05 (8) *P* > 0.05 (9) *P* < 0.05 (10) *P* < 0.05 (11) *P* > 0.05
Liang et al., 2020 (A)	SD rats (male, 20/10), NM/180-220 g	SIJ STZ (60 mg/kg)	BG ≥ 16.7 mmol/L	Artesunate, 50, 100 mg/kg/d	Same volume of NS	By intragastric, 3 weeks	(1) Bun (2) SCr (3) KI (4) TGF-*β*1↓ (5) SMAD↓ (6) AMPK↑ (7) LC3 II↑ (8) Renal pathology	(1) *P* < 0.05 (2) *P* < 0.05 (3) *P* < 0.05 (4) *P* < 0.05 (5) *P* < 0.05 (6) *P* < 0.05 (7) *P* < 0.05
Liang et al., 2020 (B)	SD rats (male, 31/10), 8 weeks/200-220 g	SIJ STZ (55 mg/kg)	BG > 16.7 mmol/L; microalbuminuria ≥ 30 mg/L	Dihydroartemisinin, 10, 20, 40 g/kg/d	Same volume of NS	By intragastric, 8 weeks	(1) Bun (2) SCr (3) Microalbuminuria (4) Body weight (5) TGF-*β*1↓ (6) SMAD3↓ (7) p- SMAD3↓ (8) Fibronectin↓ (9) E-cadherin↑	(1) *P* < 0.05 (2) *P* < 0.05 (3) *P* < 0.05 (4) *P* < 0.05 (5) *P* < 0.05 (6) *P* < 0.05 (7) *P* < 0.05 (8) *P* < 0.05 (9) *P* < 0.05
Lin, 2018	SD rats (male, 30/10), NM/180-220 g	HFD + SIJ STZ (30 mg/kg) + excision of right kidney	BG > 16.7 mmol/L	Artemisinin suspended in 0.5% sodium CMC, 25, 50, and 75 mg/kg/d	NS, 10 ml/kg/d	By intragastric, 12 weeks	(1) Proteinuria (2) Body weight (3) FPG (4) KI (5) TGF-*β*1↓ (6) RhoA↓ (7) ROCK1↓ (8) *α*-SMA↓ (9) E-cadherin↑ (10) Renal pathology	(1) *P* < 0.05 (2) *P* < 0.05 (3) *P* < 0.05 (4) *P* < 0.05 (5) *P* < 0.05 (6) *P* < 0.05 (7) *P* < 0.05 (8) *P* < 0.05 (9) *P* < 0.05
Nie et al., 2015 (A)	SD rats (male, 18/6), 6–8 weeks/180-220 g	HFD + SIJ STZ (40 mg/kg)	BG ≥ 16.7 mmol/L; urine volume > 150% of the original volume; protein excretion > 30 mg/24 h	Artesunate, 10, 20, and 30 mg/kg/d	NM	By intragastric, 8 weeks	(1) Bun (2) SCr (3) Proteinuria (4) Body weight (5) FPG (6) KI (7) MCP-1↓ (8) TNF-*α*↓ (9) TLR4↓ (10) Renal pathology	(1) *P* < 0.01 (2) *P* < 0.01 (3) *P* < 0.01 (4) *P* < 0.05 (5) *P* > 0.05 (6) *P* < 0.05 (7) *P* < 0.05 (8) *P* < 0.05 (9) *P* < 0.05
Nie et al., 2015 (B)	SD rats (male, 18/6), 6–8 weeks/180-220 g	HFD + SIJ STZ (40 mg/kg)	BG ≥ 16.7 mmol/L; urine volume > 150% of the original volume; protein excretion > 30 mg/24 h	Artesunate, 10, 20, and 30 mg/kg/d	NM	By intragastric, 8 weeks	(1) Bun (2) SCr (3) Proteinuria (4) FPG (5) MCP-1↓ (6) TNF-*α*↓ (7) Renal pathology	(1) *P* < 0.01 (2) *P* < 0.01 (3) *P* < 0.01 (4) *P* < 0.05 (5) *P* < 0.01 (6) *P* < 0.01
Nie et al., 2015 (C)	SD rats (male, 16/8), 6–8 weeks/180-220 g	HFD + SIJ STZ (30 mg/kg)	BG > 16.7 mmol/L; urine volume > 150% of the original volume; protein excretion > 30 mg/24 h	Artesunate, 10 and 30 mg/kg/d	NS, 10 mg/kg/d	By intragastric, 12 weeks	(1) Bun (2) SCr (3) Proteinuria (4) Body weight (5) FPG (6) KI (7) IL-8↓ (8) TLR4↓	(1) *P* < 0.05 (2) *P* < 0.05 (3) *P* < 0.05 (4) *P* < 0.05 (5) *P* > 0.05 (6) *P* < 0.05 (7) *P* < 0.05 (8) *P* < 0.05
Tan, 2014	SD rats (male,20/10), NM/NM	HFD + SIJ STZ (55 mg/kg) + excision of right kidney	BG ≥ 16.7 mmol/L	Artesunate, 25 and 50 mg/kg/d	Same volume of distilled water	By intragastric, 6 weeks	(1) Microalbuminuria (2) FPG (3) KI (4) TGF-*β*1↓ (5) CTGF↓ (6) Renal pathology	(1) *P* < 0.01 (2) *P* > 0.05 (3) *P* < 0.01 (4) *P* < 0.01 (5) *P* < 0.01
Wang et al., 2019	C57BL/6J mice (male,6/6), 7-8 weeks/22-26 g	Continuous intraperitoneal injections STZ (55 mg/kg)	NM	Regular diet supplemented with artemether, 670 mg/kg/d	Regular diet	By free access to the diets, 8 weeks	(1) Protein excretion (2) FPG (3) p-p27^kip^↓ (4) p-MEK1/2↓ (5) p-Erk1/2↓ (6) p-Akt↓ (7) p-mTOR↓ (8) p70S6K↓ (9) S6RP↓ (10) Catalase↑ (11) SOD2↑ (12) PDK1↓ (13) PGC-1*α*↑ (14) MPC1↑ (15) PDH↑ (16) Renal pathology	(1) *P* < 0.001 (2) *P* < 0.001 (3) *P* < 0.05 (4) *P* > 0.05 (5) *P* < 0.05 (6) *P* < 0.05 (7) *P* < 0.05 (8) *P* < 0.01 (9) *P* < 0.05 (10) *P* < 0.01 (11) *P* > 0.05 (12) *P* > 0.05 (13) *P* > 0.05 (14) *P* < 0.05 (15) *P* > 0.05 (16) *P* < 0.05
Xiang et al., 2019	SD rats (male, 6/6), NM/250-300 g	SIJ STZ (60 mg/kg)	BG > 16.6 mmol/L; urine volume > 150% of the original volume; proteinuria > 200% before modeling	Artemisinin, 300 mg/kg/d	Same volume of NS	By intragastric, 4 weeks	(1) Bun (2) SCr (3) Proteinuria (4) FPG (5) ID1↓ (6) IGFBP1↑ (7) Cdkn1a↓ (8) HMGCS2↓ (9) Rarres2↓ (10) Sult1a1↑ (11) Pigr↑ (12) Steap4↑ (13) Renal pathology	(1) *P* < 0.05 (2) *P* < 0.05 (3) *P* < 0.05 (4) *P* < 0.05 (5) *P* > 0.01 (6) *P* < 0.01 (7) *P* < 0.01 (8) *P* < 0.01 (9) *P* < 0.05 (10) *P* < 0.01 (11) *P* > 0.01 (12) *P* < 0.01
Yang and Zhang, 2017	Wistar rats (male, 40/20), NM/200-260 g	HFD + SIJ STZ (30 mg/kg)	BG > 16.7 mmol/L	Artesunate, 10 and 30 mg/kg/d	NS, 1 ml/kg/d	By intragastric, 8 weeks	(1) Bun (2) SCr (3) Proteinuria (4) Body weight (5) FPG (6) KI (7) IL-8↓ (8) TLR4↓	(1) *P* < 0.05 (2) *P* < 0.05 (3) *P* < 0.05 (4) *P* < 0.05 (5) *P* > 0.05 (6) *P* < 0.05 (7) *P* < 0.05 (8) *P* < 0.05
Zhang et al., 2014 (A)	SD rats (male, 6/6), NM/180-220 g	SIJ STZ (65 mg/kg)	BG ≥ 16.7 mmol/L	Artemisinin suspended in DMSO, 75 mg/kg/d	Same volume of DMSO	By SIJ, 6 weeks	(1) Bun (2) SCr (3) Protein excretion (4) Body weight (5) FPG (6) KI (7) PDGF-B↓ (8) Renal pathology	(1) *P* < 0.05 (2) *P* < 0.05 (3) *P* < 0.05 (4) *P* < 0.05 (5) *P* > 0.05 (6) *P* < 0.05 (7) *P* < 0.05
Zhang et al., 2014 (B)	SD rats (male, 6/6), NM/180-220 g	SIJ STZ (65 mg/kg)	BG ≥ 16.7 mmol/L	Artemisinin suspended in DMSO, 75 mg/kg/d	Same volume of DMSO	By SIJ, 6 weeks	(1) Bun (2) SCr (3) Protein excretion (4) Body weight (5) FPG (6) KI (7) PKC↓ (8) Renal pathology	(1) *P* < 0.05 (2) *P* < 0.05 (3) *P* < 0.05 (4) *P* < 0.05 (5) *P* > 0.05 (6) *P* < 0.05 (7) *P* < 0.05
Zhang et al., 2014 (C)	SD rats (male, 6/6), NM/180-220 g	SIJ STZ (65 mg/kg)	BG ≥ 16.7 mmol/L	Artemisinin suspended in DMSO, 75 mg/kg/d	Same volume of DMSO	By SIJ, 6 weeks	(1) Bun (2) SCr (3) Protein excretion (4) Body weight (5) FPG (6) KI (7) MMP-2↑ (8) TIMP-2↓ (9) Fibronectin↓ (10) Col4A1↓ (11) Renal pathology	(1) *P* < 0.05 (2) *P* < 0.05 (3) *P* < 0.05 (4) *P* < 0.05 (5) *P* > 0.05 (6) *P* < 0.05 (7) *P* < 0.05 (8) *P* < 0.05 (9) *P* < 0.05 (10) *P* < 0.05
Zhang et al., 2020	SD rats (male, 33/10), 5-6 weeks/160-200 g	SIJ STZ (55 mg/kg)	BG > 16.7 mmol/L	Artemisinin suspended in 0.5% sodium CMC, 25, 50, and 75 mg/kg/d	Same volume of 0.5% SCC	By intragastric, 8 weeks	(1) Bun (2) SCr (3) Protein excretion (4) Body weight (5) FPG (6) KI (7) TGF-*β*1↓ (8) Nrf2↑ (9) HO-1↑ (10) NQO-1↑ (11) MDA↓ (12) T-SOD↑ (13) GSH-Px↑ (14) Renal pathology	(1) *P* < 0.05 (2) *P* < 0.05 (3) *P* < 0.05 (4) *P* < 0.05 (5) *P* < 0.05 (6) *P* < 0.05 (7) *P* < 0.05 (8) *P* < 0.05 (9) *P* < 0.05 (10) *P* < 0.05 (11) *P* < 0.05 (12) *P* < 0.05 (13) *P* < 0.05
Zhou et al., 2014 (A)	SD rats (male, 5/5), NM/180-220 g	SIJ STZ (65 mg/kg)	BG ≥ 16.7 mmol/L	Artemisinin suspended in DMSO, 300 mg/kg/d	Same volume of DMSO	By SIJ, 4 weeks	(1) AP-1↓ (2) Renal pathology	(1) *P* < 0.01
Zhou et al., 2014 (B)	SD rats (male, 5/5), NM/180-220 g	SIJ STZ (65 mg/kg)	BG ≥ 16.7 mmol/L	Artemisinin suspended in DMSO, 300 mg/kg/d	Same volume of DMSO	By SIJ, 4 weeks	(1) c-Jun↓ (2) c-Fos↓ (3) Renal pathology	(1) *P* < 0.01 (2) *P* < 0.01
Zhou et al., 2014 (C)	SD rats (male, 5/5), NM/180-220 g	SIJ STZ (65 mg/kg)	BG ≥ 16.7 mmol/L	Artemisinin suspended in DMSO, 300 mg/kg/d	Same volume of DMSO	By SIJ, 4 weeks	(1) NF-*κ*B↓ (2) Renal pathology	(1) *P* < 0.01

Abbreviations: AMPK: AMP-activated protein kinase; BG: blood glucose; BUN: blood urea nitrogen; Cdkn1a: cyclin dependent kinase inhibitor 1A; Col4A1: collagen type IV alpha-1 chain; CTGF: connective tissue growth factor; DMSO: dimethyl sulfoxide; Erk: extracellular signal-regulated kinase; FPG: fasting plasma glucose; GSH-Px: glutathione peroxidase; HFD: high-fat diet; Hmgcs2: 3-hydroxy-3-methylglutaryl-CoA synthase 2; HO-1: heme oxygenase-1; Id1: inhibitor of DNA binding 1; Igfbp1: insulin like growth factor binding protein 1; IL-8: interleukin-8; KI: kidney index; LC3 II: microtubule-associated proteins 1A/1B light chain 3B type II; MAPK: mitogen-activated protein kinase; MCP-1: monocyte chemoattractant protein-1; MDA: malondialdehyde; MPC1 & MPC2: mitochondrial pyruvate carrier 1 & 2; MMP-2: matrix metallopeptidase-2; NF-*κ*B: nuclear factor kappa B; NM: not mentioned; NQO-1: NAD(P)H quinone oxidoreductase-1; Nrf2: nuclear factor erythroid 2-related factor 2; NS: normal saline; p-Akt: phospho-protein kinase B; p-mTOR: phospho-mammalian target of rapamycin; PGC-1*α*: peroxisome proliferator-activated receptor *γ* coactivator-1*α*; PDGF-B: platelet-derived growth factor-B; PDH: pyruvate dehydrogenase; PDP1:pyruvate dehydrogenase phosphatase 1; PDK1: pyruvate dehydrogenase kinase 1; Pigr: polymeric immunoglobulin receptor; PKC: protein kinase C; Rarres2: retinoic acid receptor responder 2; RhoA: ras-homolog gene family, member A; ROCK1: rho-associated coiled-coil containing protein kinase 1; SCr: serum creatinine; SD rats: Sprague Dawley rats; SIJ: single intraperitoneal injection; SOD: superoxide dismutase; *α*-SMA: *α*-smooth muscle actin; STZ: streptozotocin; sodium CMC: sodium carboxymethyl cellulose; Sult1a1: sulfotransferase 1A1; Steap4: six-transmembrane epithelial antigen of prostate 4; TGF-*β*1: transforming growth factor-*β*1; TIMP-2: tissue inhibitor of metalloproteinase-2; TLR4: toll-like receptor 4; TNF-*α*: tumor necrosis factor-*α*; T-SOD: total superoxide dismutase. Compared with the control group, ↓ indicates reduction while ↑ indicates increase.

**Table 2:** Subgroup analyses in the association of BUN and SCr with the ex ante parameters.

Comparison	Subgroup	No. of studies	SMD [95% CI]	*P* for meta-analysis	*I* ^2^	*P* for heterogeneity
BUN						
Modeling methods	STZ	7	-2.76 [-4.09, -1.43]	<0.0001	84%	<0.00001
	STZ + HFD	4	-4.49 [-5.18, -3.80]	<0.00001	0%	0.98
Artemisinins types	Artesunate	5	-3.74 [-5.42, -2.06]	<0.0001	89%	<0.00001
	Dihydroartemisinin	1	-0.90 [-1.65, -0.16]	0.02	/	/
	Artemisinin	5	-5.43 [-8.42, -2.44]	0.0004	88%	<0.00001
Route of administration	By intragastric	8	-2.82 [-4.00, -1.64]	<0.00001	89%	<0.00001
	By SIJ	3	-9.26 [-11.97, -6.54]	<0.00001	0%	0.63
Duration of treatment	≤8 weeks	10	-3.64 [-4.98, -2.31]	<0.00001	90%	<0.00001
	>8 weeks	1	-4.32 [-5.90, -2.74]	<0.00001	/	/
SCr						
Modeling methods	STZ	7	-2.69 [-3.90, -1.47]	<0.0001	81%	<0.0001
	STZ + HFD	4	-2.83 [-3.35, -2.31]	<0.00001	0%	0.73
Artemisinins types	Artesunate	5	-2.53 [-3.23, -1.83]	<0.00001	55%	0.06
	Dihydroartemisinin	1	-0.65 [-1.37, -0.08]	0.08	/	/
	Artemisinin	5	-4.16 [-6.21, -2.10]	<0.0001	78%	0.001
Route of administration	By intragastric	8	-2.20 [-2.90, -1.50]	<0.00001	74%	0.003
	By SIJ	3	-6.10 [-9.01, -3.20]	<0.0001	56%	0.10
Duration of treatment	≤8 weeks	10	-2.76 [-3.66, -1.86]	<0.00001	80%	<0.00001
	>8 weeks	1	-2.48 [-3.63, -1.34]	<0.0001	/	/

Abbreviations: BUN: blood urea nitrogen; CI: confidence interval; HFD: high-fat diet; SCr: serum creatinine; SIJ: single intraperitoneal injection; SMD: standard mean difference; STZ: streptozotocin.

**Table 3:** Results from Egger's test and trim and fill analysis on BUN and SCr.

Outcomes	Egger's *P* value	Before trim and fill			After trim and fill		
		*P* value	Total effect sizes [95% CI]	No. studies	*P* value	Total effect sizes [95% CI]	No. studies
BUN	0.002	<0.00001	-3.71 [-4.98, -2.45]	11	0.005	-1.984 [-3.369, -0.599]	16
SCr	0.002	<0.00001	-2.70 [-3.50, -1.89]	11	<0.0001	-1.924 [-2.808,-1.047]	16

Abbreviations: BUN: blood urea nitrogen; CI: confidence interval; SCr: serum creatinine.

## Data Availability

The data used to support the findings of this study are available from the corresponding author upon request.

## Supplementary Materials

Supplementary 1 Table S1: the methodological quality of included studies.

Supplementary 2 Item S1: search strategy for PubMed.

Supplementary 3 Figure S2: forest plots for subgroup analysis.
