# Supplementary material for: Protective Effect and Possible Mechanisms of Artemisinin and Its Derivatives for Diabetic Nephropathy: A Systematic Review and Meta-Analysis in Animal Models
Source: Oxid Med Cell Longev. 2022 Apr 25;2022:5401760. doi: 10.1155/2022/5401760 (PMC9073547; doi:10.1155/2022/5401760)
Supplement: Supplementary 1 — Table S1: the methodological quality of included studies. [file 5401760.f1.docx]

**Table S1.** The methodological quality of included studies

| **Study** | **A** | **B** | **C** | **D** | **E** | **F** | **G** | **H** | **I** | **J** | **Total** |
| --- | --- | --- | --- | --- | --- | --- | --- | --- | --- | --- | --- |
| Han 2019 | + | + | ? | + | ? | - | ? | - | + | + | 5 |
| Lin 2018 | ? | ? | ? | + | ? | - | ? | + | + | - | 3 |
| Liang 2020(A) | ? | ? | ? | + | ? | - | ? | + | + | + | 4 |
| Liang 2020(B) | ? | ? | ? | ? | ? | - | ? | + | + | + | 3 |
| Nie 2015 (A) | + | + | ? | ? | ? | - | ? | + | + | + | 5 |
| Nie 2015 (B) | ? | ? | ? | ? | ? | - | ? | + | + | + | 3 |
| Nie 2015 (C) | + | + | ? | ? | ? | - | ? | + | + | + | 5 |
| Tan 2014 | ? | ? | ? | ? | ? | - | ? | + | + | - | 2 |
| Wang 2019 | ? | + | ? | + | ? | - | ? | + | + | + | 5 |
| Xiang 2019 | ? | ? | ? | + | ? | - | ? | + | + | + | 4 |
| Yang 2017 | ? | ? | ? | + | ? | - | ? | + | + | + | 4 |
| Zhang 2014 (A) | ? | ? | ? | ? | ? | - | ? | + | + | + | 3 |
| Zhang 2014 (B) | ? | ? | ? | ? | ? | - | ? | + | + | + | 3 |
| Zhang 2014 (C) | ? | ? | ? | ? | ? | - | ? | + | + | + | 3 |
| Zhang 2020 | ? | ? | ? | ? | ? | - | ? | - | + | + | 2 |
| Zhou 2014 (A) | ? | ? | ? | ? | ? | - | ? | + | + | + | 3 |
| Zhou 2014 (B) | ? | ? | ? | ? | ? | - | ? | + | + | + | 3 |
| Zhou 2014 (C) | ? | ? | ? | ? | ? | - | ? | + | + | + | 3 |

Note: Selection bias: A, Sequence generation; B, Baseline characteristics; C, Allocation concealment. Performance bias: D, Random housing; E, Blinding. Detection bias: F, Random outcome assessment; G, Blinding. Attrition bias: H, Incomplete outcome data. Reporting bias: I, Selective outcome reporting. Other: J, Other sources of bias. ?, unclear; +, low risk; -, high risk.
