# Supplementary material for: Protective Effect and Possible Mechanisms of Artemisinin and Its Derivatives for Diabetic Nephropathy: A Systematic Review and Meta-Analysis in Animal Models
Source: Oxid Med Cell Longev. 2022 Apr 25;2022:5401760. doi: 10.1155/2022/5401760 (PMC9073547; doi:10.1155/2022/5401760)
Supplement: Supplementary 2 — Item S1: search strategy for PubMed. [file 5401760.f2.docx]

**Item S1.** Search strategy for PubMed

| Number | Search terms |
| --- | --- |
| 1 | “Animals” [Mesh Terms] OR “Models, Animal” [Mesh Terms] OR “Animals, Laboratory” [Mesh Terms] OR “Animal Experimentation” [Mesh Terms] |
| 2 | “Animalia” [Title/Abstract] OR “Animal” [Title/Abstract] OR “Metazoa” [Title/Abstract] OR “Animal Model” [Title/Abstract] OR “Animal Models” [Title/Abstract] OR “Model, Animal” [Title/Abstract] OR “Laboratory Animal Models” [Title/Abstract] OR “Animal Model, Laboratory” [Title/Abstract] OR “Animal Models, Laboratory” [Title/Abstract] OR “Laboratory Animal Model” [Title/Abstract] OR “Model, Laboratory Animal” [Title/Abstract] OR “Experimental Animal Models” [Title/Abstract] OR “Animal Model, Experimental” [Title/Abstract] OR “Animal Models, Experimental” [Title/Abstract] OR “Experimental Animal Model” [Title/Abstract] OR “Model, Experimental Animal” [Title/Abstract] OR “Models, Experimental Animal” [Title/Abstract] OR “Experimentation, Animal” [Title/Abstract] OR “Animal Research” [Title/Abstract] OR “Research, Animal” [Title/Abstract] OR “Animal Experimental Use” [Title/Abstract] OR “Animal Experiments” [Title/Abstract] OR “Animal Experiment” [Title/Abstract] OR “Experiment, Animal” [Title/Abstract] OR “Experiments, Animal” [Title/Abstract] OR “Animal, Laboratory” [Title/Abstract] OR “Laboratory Animal” [Title/Abstract] OR “Laboratory Animals” [Title/Abstract] OR “preclinical studies” [Title/Abstract] OR “experimental animals” [Title/Abstract] OR “experimental animal” [Title/Abstract] |
| 3 | 1 OR 2 |
| 4 | “Diabetic Nephropathies” [Mesh Terms] |
| 5 | “Nephropathies, Diabetic” [Title/Abstract] OR “Nephropathy, Diabetic” [Title/Abstract] OR “Diabetic Nephropathy” [Title/Abstract] OR “Diabetic Kidney Disease” [Title/Abstract] OR “Diabetic Kidney Diseases” [Title/Abstract] OR “Kidney Disease, Diabetic” [Title/Abstract] OR “Kidney Diseases, Diabetic” [Title/Abstract] OR “Diabetic Glomerulosclerosis” [Title/Abstract] OR “Glomerulosclerosis, Diabetic” [Title/Abstract] OR “Intracapillary Glomerulosclerosis” [Title/Abstract] OR “Nodular Glomerulosclerosis” [Title/Abstract] OR “Glomerulosclerosis, Nodular” [Title/Abstract] OR “Kimmelstiel-Wilson Syndrome” [Title/Abstract] OR “Kimmelstiel Wilson Syndrome” [Title/Abstract] OR “Syndrome, Kimmelstiel-Wilson” [Title/Abstract] OR “Kimmelstiel-Wilson Disease” [Title/Abstract] OR “Kimmelstiel Wilson Disease” [Title/Abstract] OR DN [Title/Abstract] OR DKD [Title/Abstract] |
| 6 | 4 OR 5 |
| 7 | “Artesunate” [Mesh Terms] OR “Artemisinins” [Mesh Terms] OR “Artemether” [Mesh Terms] OR “Artemisia annua” [Mesh Terms] OR “artemotil” [Supplementary Concept] OR “dihydroartemisinin” [Supplementary Concept] OR “artelinic acid” [Supplementary Concept] OR “artemisinine” [Supplementary Concept] |
| 8 | “Succinyl Dihydroartemisinin” [Title/Abstract] OR “Dihydroartemisinin, Succinyl” [Title/Abstract] OR Dihydroartemisinine-12-alpha-succinate [Title/Abstract] OR “Dihydroartemisinine 12 alpha succinate” [Title/Abstract] OR Malacef [Title/Abstract] OR “Sodium Artesunate” [Title/Abstract] OR Malartin [Title/Abstract] OR SM-804 [Title/Abstract] OR “SM 804” [Title/Abstract] OR “SM804” [Title/Abstract] OR O-Methyldihydroartemisinine [Title/Abstract] OR “O Methyldihydroartemisinine” [Title/Abstract] OR Artenam [Title/Abstract] OR beta-Arthemeter [Title/Abstract] OR “beta Arthemeter” [Title/Abstract] OR “Artemether, 3R-3alpha,5abeta,6beta,8abeta,9alpha,10alpha,12beta,12aR*-isomer” [Title/Abstract] OR “Artemether, 3R-3alpha,5abeta,6alpha,8abeta,9alpha,10beta,12beta,12aR*-isomer” [Title/Abstract] OR alpha-Artemether [Title/Abstract] OR “alpha Artemether” [Title/Abstract] OR “Artemether, 3R-3alpha,5abeta,6beta,8aalpha,9alpha,10beta,12beta,12aR*-isomer” [Title/Abstract] OR “quinghaosu, dihydro-” [Title/Abstract] OR dihydroartemisinine [Title/Abstract] OR dihydroqinghaosu [Title/Abstract] OR dihydroquinghaosu [Title/Abstract] OR “artemisinin, dihydro-” [Title/Abstract] OR “3alpha-hydroxydeoxydihydroartemisinin” [Title/Abstract] OR 8alpha-hydroxydeoxyartemisinin [Title/Abstract] OR 9alpha-hydroxydeoxyartemisinin [Title/Abstract] OR “dihydroquinghaosu, 3R-3alpha,5abeta,6beta,8abeta,9alpha,10alpha,12alpha,12aR*-isomer” [Title/Abstract] OR “dihydroquinghaosu, 3R-3alpha,5abeta,6beta,8abeta,9alpha,10beta,12alpha,12aR*-isomer” [Title/Abstract] OR artether [Title/Abstract] OR arteether [Title/Abstract] OR SM-227 [Title/Abstract] OR beta-artether [Title/Abstract] OR “arteether, 3R-3alpha,5abeta,6beta,8abeta,9alpha,10alpha,12beta,12aR*-isomer” [Title/Abstract] OR “arteether, 3R-3alpha,5abeta,6beta,8abeta,9alpha,10beta,12beta,12aR*-isomer” [Title/Abstract] OR Qinghao [Title/Abstract] OR “Sweet Wormwood” [Title/Abstract] OR “Sweet Wormwoods” [Title/Abstract] OR “ Wormwood, Sweet “ [Title/Abstract] OR “ Wormwoods, Sweet “ [Title/Abstract] OR “Qing Hao” [Title/Abstract] OR “Sweet Annie” [Title/Abstract] OR “Sweet Annies” [Title/Abstract] OR “Annie, Sweet” [Title/Abstract] OR “Annies, Sweet” [Title/Abstract] OR SESQUITERPENES [Title/Abstract] OR “artelinic acid, potassium salt” [Title/Abstract] OR “artelinic acid, 3R-3alpha,5beta,6beta,8abeta,9alpha,10alpha,12beta,12aR*-isomer” [Title/Abstract] OR “sodium beta-artelinate” [Title/Abstract] OR “sodium artelinate” [Title/Abstract] OR artemisinin [Title/Abstract] OR “quing hau sau” [Title/Abstract] OR quinghaosu [Title/Abstract] OR arteannuin [Title/Abstract] OR qinghaosu [Title/Abstract] |
| 9 | 7 OR 8 |
| 10 | 3 AND 6 AND 9 |
