# Supplementary material for: Protective Effect and Possible Mechanisms of Artemisinin and Its Derivatives for Diabetic Nephropathy: A Systematic Review and Meta-Analysis in Animal Models
Source: Oxid Med Cell Longev. 2022 Apr 25;2022:5401760. doi: 10.1155/2022/5401760 (PMC9073547; doi:10.1155/2022/5401760)
Supplement: Supplementary 3 — Figure S2: forest plots for subgroup analysis. [file 5401760.f3.docx]

**(a)**
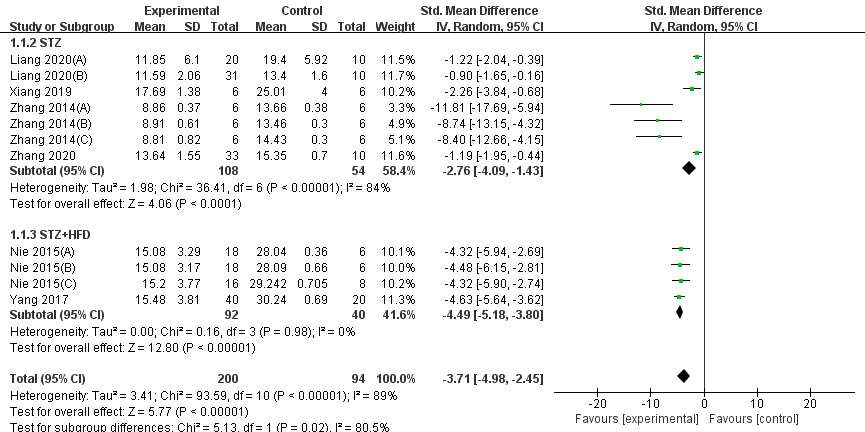


**(b)**
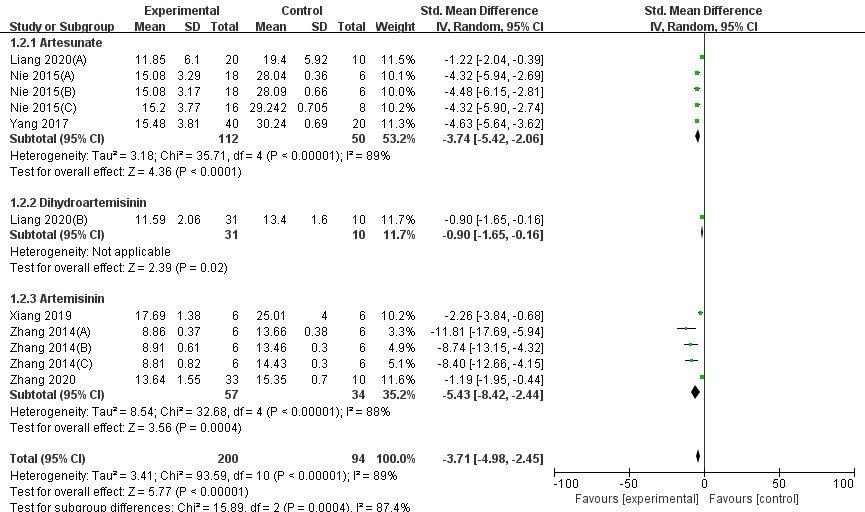


**(c)**
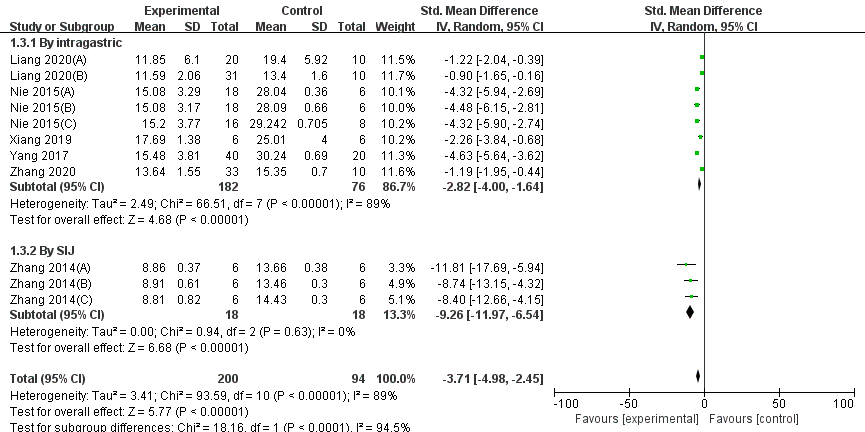


**(d)**
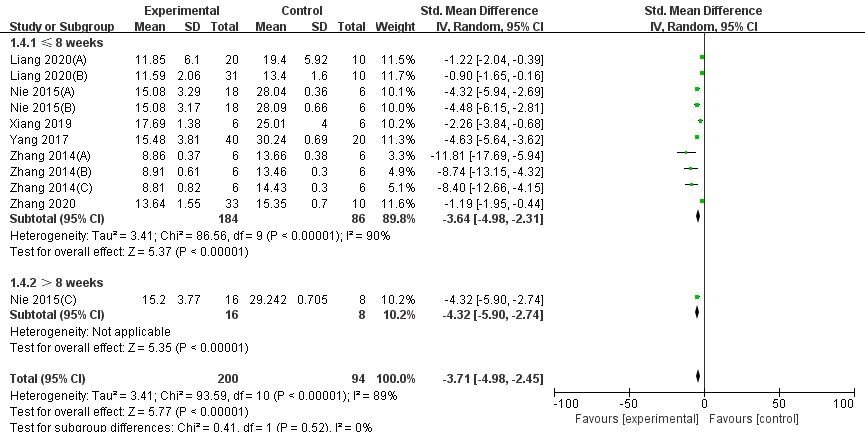


**Figure S1.** Effects of artemisinins on blood urea nitrogen (BUN) in animals with diabetic kidney disease (DKD) compared with vehicle control by the subgroup of different (a) modeling methods, (b) artemisinins types, (c) route of administration and (d) duration of treatment. Abbreviations: BUN, blood urea nitrogen; CI, confidence interval; HFD, high-fat diet; IV, inverse variance; SD, standard deviation; SIJ: single intraperitoneal injection; STZ: streptozotocin.

**(a)**
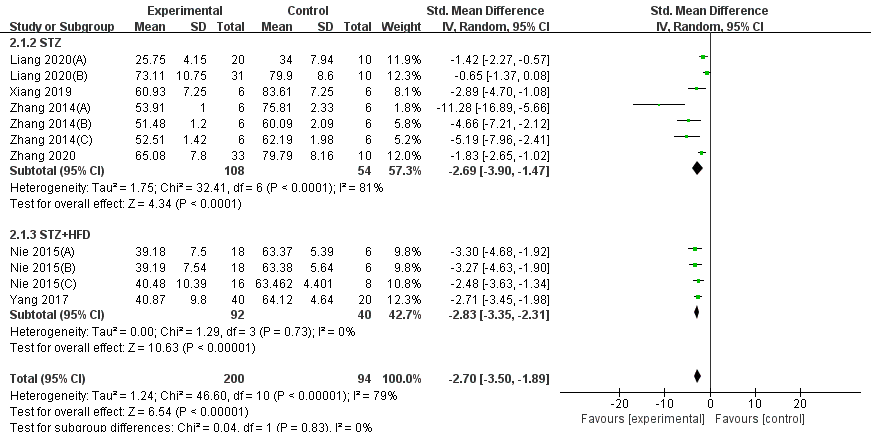


**(b)**
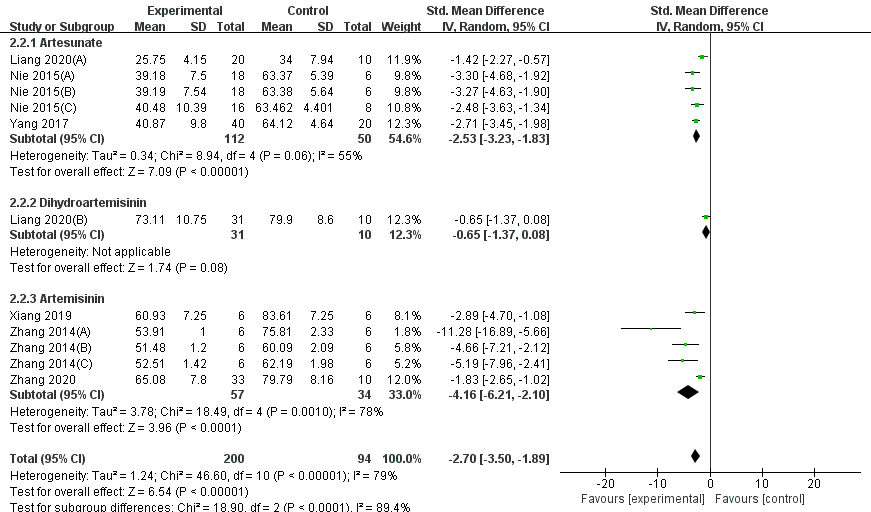


**(c)**
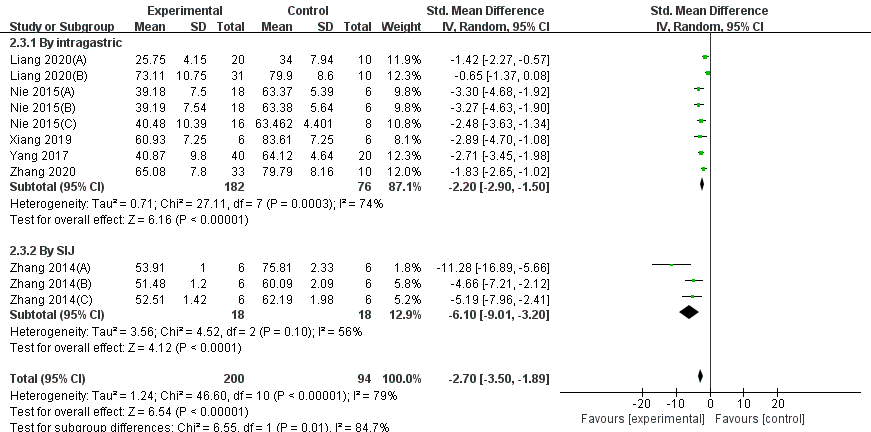


**(d)**
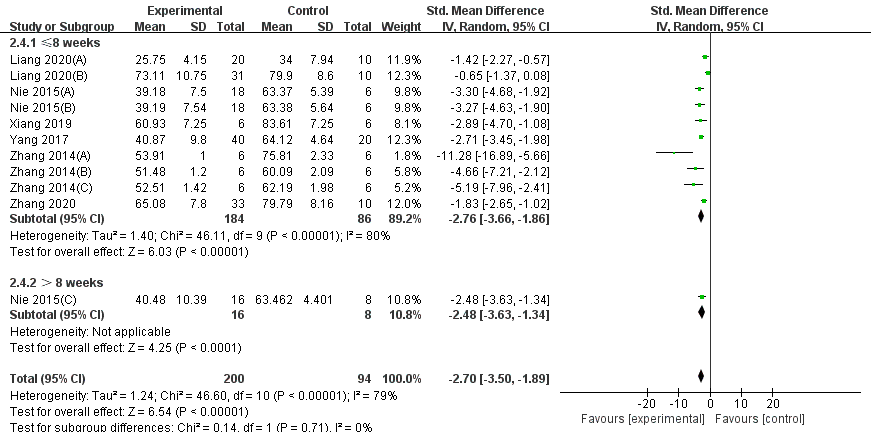


**Figure S2.** Effects of artemisinins on serum creatinine (SCr) in animals with diabetic kidney disease (DKD) compared with vehicle control by the subgroup of different (a) modeling methods, (b) artemisinins types, (c) route of administration and (d) duration of treatment. Abbreviations: CI, confidence interval; HFD, high-fat diet; IV, inverse variance; SCr: serum creatinine; SD, standard deviation; SIJ: single intraperitoneal injection; STZ: streptozotocin.


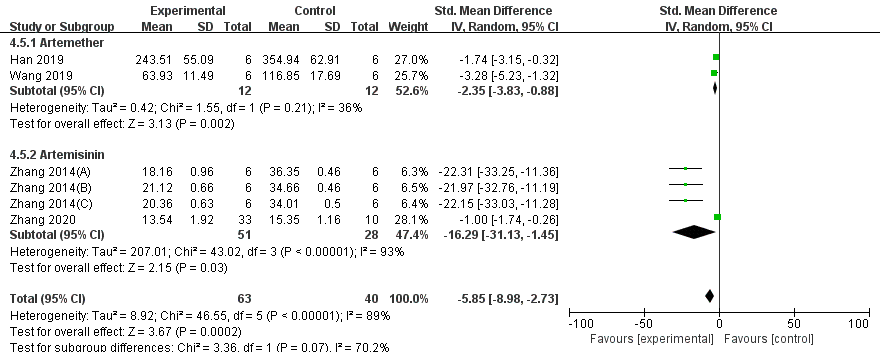


**Figure S3.** Effects of artemisinins on urinary protein excretion in animals with daibetic kidney disease (DKD) compared with vehicle control by the subgroup of different artemisinins types. Abbreviations: CI, confidence interval; IV, inverse variance; SD, standard deviation.
